# Potential Effects of Low-Calorie Sweeteners on Human Health

**DOI:** 10.3390/nu17172726

**Published:** 2025-08-22

**Authors:** Huang-Pin Chen, Yuan Kao, Meng-Wei Lin, Chun-Te Lee, Hung-Tsung Wu, Hsin-Yu Kuo

**Affiliations:** 1 Division of Gastroenterology and Hepatology, Department of Internal Medicine, National Cheng Kung University Hospital, College of Medicine, National Cheng Kung University, Tainan 701, Taiwan; n108108@mail.hosp.ncku.edu.tw (H.-P.C.); n104893@mail.hosp.ncku.edu.tw (M.-W.L.); n048074@mail.hosp.ncku.edu.tw (C.-T.L.); 2 Department of Emergency Medicine, Chi Mei Medical Center, Tainan 710, Taiwan; 950162@mail.chimei.org.tw; 3 Department of Nursing, National Tainan Junior College of Nursing, Tainan 700, Taiwan; 4 Department of Internal Medicine, School of Medicine, College of Medicine, National Cheng Kung University, Tainan 701, Taiwan; 5 Tong-Yuan Diabetes Center, College of Medicine, National Cheng Kung University, Tainan 701, Taiwan

**Keywords:** artificial sweetener, aspartame, diabetes, obesity, sucralose

## Abstract

Low-calorie sweeteners (LCS) are widely utilized as sugar substitutes due to their intense sweetness, thermal stability, and applicability in weight management and diabetic-friendly products. However, increasing evidence has raised concerns about their potential long-term effects on metabolic health, glucose regulation, cardiovascular function, carcinogenicity, and gut microbiota composition. This review systematically evaluates the pharmacokinetics, metabolic effects, and associated health outcomes of major LCS. Mechanistically, LCS exert effects via sweet taste receptor-mediated pathways, altering glucose absorption, insulin secretion, and intracellular signaling cascades. Additionally, LCS influence gut microbiota composition, with certain agents promoting dysbiosis and glucose intolerance. While some findings support the metabolic benefits of selected LCS, others underscore potential risks, necessitating cautious interpretation. In conclusion, while LCS offer viable alternatives to sugar, their health effects are context-dependent and may vary across different sweeteners and populations. Long-term, high-quality clinical trials are essential to elucidate their safety and efficacy.

## 1. Introduction

The global prevalence of diabetes has reached alarming levels. According to the International Diabetes Federation, approximately 589 million adults aged 20–79 were living with diabetes in 2025, and this number is projected to rise to 853 million by 2050 if no effective preventive measures are implemented. The economic burden associated with diabetes is substantial. In the United States alone, diabetes-related healthcare expenditures exceeded USD 412.9 billion in 2022, a significant increase from USD 327 billion in the previous year. On a global scale, the total annual cost of diabetes management is estimated to exceed USD 760 billion. In response to the rising prevalence of diabetes and obesity, the global low-calorie sweetener market is expanding rapidly. Valued at USD 10–33 billion in 2025, it is projected to grow at a compound annual growth rate (CAGR) of 4.7–6.0%, reaching USD 14–52 billion by 2030–2035. Sucralose leads the market, while natural sweeteners like stevia are gaining momentum. These trends underscore the importance of understanding the metabolic effects of low-calorie sweeteners.

Low-calorie sweeteners (LCS) have become common substitutes to sucrose in modern diets, offering sweetness with fewer or no calories. Among these, substances can generally be divided into non-caloric artificial sweeteners, such as sucralose, acesulfame potassium (AceK), aspartame, and natural low-calorie sweeteners, including stevioside, and sorbitol. These sweeteners have gained widespread use in various food and beverage products due to their unique properties, including intense sweetness, stability under heat, and suitability for diabetic or weight management diets. However, concerns about their long-term health effects, including the impacts on glucose metabolism, body weight control, cardiovascular health, and potential carcinogenicity, have sparked significant scientific and public interest. Thus, we review the characteristics, benefits, and potential health risks associated with LCS to provide a comprehensive understanding of their role in dietary choices and public health.

**Figure 1:**
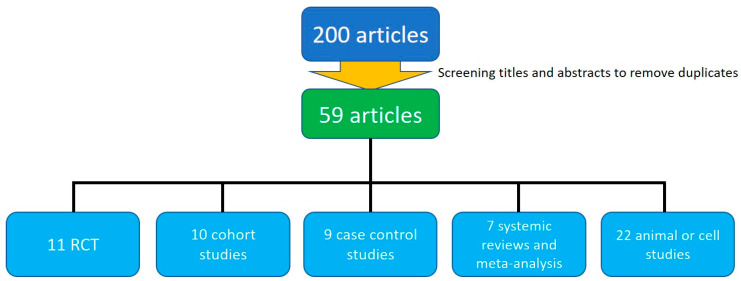
Study searching.

We conducted the literature search in accordance with the PRISMA 2020 guidelines; however, since this review primarily aims to provide a broad synthesis of key aspects of commonly used artificial sweeteners, certain adjustments were made to the standard process. A total of 200 records were identified from electronic databases and additional sources, e.g., PubMed, Embase, and Cochrane, with key words of sucralose, aspartame, acesulfame potassium (AceK), stevioside, erythritol, xylitol, sorbitol, maltitol, saccharin, blood glucose, body weight, lipid, and disease risks. After removing duplicates and screening titles and abstracts, 59 full-text articles were assessed for eligibility. We subsequently categorized the retrieved articles by different types of artificial sweeteners and discussed them accordingly. The selection process is detailed in the diagram (Figure 1).

We have organized the relevant studies on various low-calorie sweeteners (LCS) into structured summary tables (Table 1, Table 2, Table 3, Table 4, Table 5, Table 6, Table 7, Table 8 and Table 9), using the categories Category, Measured parameter, Conclusion, Author, and Year [ref.], to provide a concise and comparative overview of the key findings related to each sweetener. The “Measured parameter” column has been standardized to indicate the primary outcome or focus of each study (e.g., glycemic responses, body weight, serum lipid profile, disease risks), which corresponds directly to the structure and content of the respective sub-sections in the main text.

### 1.1. Sucralose

Sucralose is a commonly used artificial sweetener that is 600-fold sweeter than sucrose and is stable under heat. It is found in various products, including tabletop sweeteners and baked goods. The acceptable daily intake (ADI) of sucralose is set at 5 mg per kilogram of body weight per day by regulatory agencies, such as the U.S. Food and Drug Administration (FDA) and the European Food Safety Authority (EFSA) [1]. Sucralose is poorly absorbed in the small intestine, with about 15–27% entering the bloodstream, while the majority (~85%) passes through the digestive tract unchanged [2]. The absorbed portion is excreted unchanged via the kidneys. In addition, high doses may influence gut microbiota, but normal consumption levels (within safety limits) have minimal impact [3,4]. As shown in Table 1, we summarized the possible effects of sucralose on human health.

In several fundamental experimental studies, sucralose has been shown to exert measurable effects on metabolic processes. In mice, it has been associated with increased hepatic steatosis when combined with a high-fat diet [5]. Similarly, in rats, high-dose sucralose administration (4 g/kg/day) resulted in elevated triglyceride, cholesterol, and low-density lipoprotein cholesterol (LDL-c) levels, accompanied by marked hepatic histopathological alterations, including hemorrhagic lesions, chromatin condensation and loss, hepatocellular vacuolation, and infiltration of inflammatory cells [6]. Furthermore, another animal study demonstrated that long-term exposure to sucralose in drinking water for 12 weeks at varying concentrations (0.27, 0.33, 0.40, and 0.47 g/L) induced weight gain, impaired glucose homeostasis, and elevated blood glucose levels, potentially mediated through the upregulation of intestinal sweet taste receptors and glucose transporters [7,8].

In clinical investigations, similar phenomena have also been documented (Table 1). Although the substitution of sugar with LCS has traditionally been considered supportive of weight control, several studies have indicated that sucralose may worsen certain health outcomes, particularly those related to glycemic regulation [9]. In a controlled trial involving 45 healthy adults and a smaller cohort of adolescents, participants consumed beverages sweetened with either sucralose alone (LCS group), sucrose (Sugar group), or a combination of sucralose and maltodextrin (Combo group) over a two-week period, corresponding to a total of seven drinks and an average daily sucralose intake of approximately 0.43 mg/kg. The results showed that sucralose consumption reduced insulin sensitivity and altered the gut microbiota, potentially contributing to impaired glycemic responses [8,10]. By contrast, another short-term study in 17 healthy adults (10 females and 7 males) reported that daily supplementation with 136 mg of sucralose (~20% of the ADI) for two weeks did not significantly affect glucose metabolism [11].

The above findings from human and animal studies suggest sucralose may impair insulin sensitivity, alter gut microbiota, and affect liver and lipid metabolism, particularly at high doses or with long-term use. Regarding cardiovascular diseases, a study was conducted on 103,388 healthy participants in the web-based NutriNet cohort. Three non-consecutive days of 24 h dietary records were randomly collected over a two week period for two years that examined the associations among aspartame, AceK, sucralose, and cardiovascular disease risk, and it was found that total artificial sweetener intake was associated with an increased cardiovascular risk [12]. These findings highlight the need for further research to evaluate its long-term safety in typical dietary settings.

**Table 1:** Sucralose-related human studies.

Category	Measured Parameter	Conclusion	Author, Year [Ref.]
Meta-analysis	Body weight	Positive association with weight gain	Laviada-Molina et al., 2020 [9]
RCT	Glycemic responses	Impaired insulin sensitivity and dysregulated glycemic responses	Dalenberg, J.R., et al., 2020 [10]
Case control	Disease risks	Increased cardiovascular risk	Debras, C., et al., 2022 [12]

RCT, randomized controlled trial.

### 1.2. Acesulfame Potassium (AceK)

AceK is a commonly used calorie-free sweetener, and the ADI of AceK is 15 mg per kilogram of body weight per day, established by the U.S. FDA. Its high sweetness intensity, stability under heat, and low cost make it a popular choice for manufacturers, especially in dietary and sugar-free products. AceK is rapidly absorbed in the small intestine and enters the bloodstream by prototype within 30–60 min after consumption [13]. AceK is not metabolized by the liver or any other organs in the body and is rapidly filtered by the kidneys and excreted through urine. Studies showed that over 95% of ingested AceK is excreted unchanged within 24 h [14].

On the aspect of whether AceK impacts blood sugar levels, public health recommendations are conflicting. Rathaus et al. reported that chronic AceK consumption exerted no significant effects on body weight, fat mass, or glucose metabolism in mice supplemented with AceK in drinking water in combination with either standard chow or a high fat diet over a 20-week period [15]. In contrast, Lin et al. found that concurrent intake of AceK exacerbated high cholesterol diet-induced dyslipidemia and atherosclerotic lesion formation in ApoE^−/−^ mice during an 8-week study, in which animals received AceK supplementation at 15 mg/kg/day, a dose equivalent to the human ADI [16]. Furthermore, both sucralose and AceK have been reported to induce cardiotoxic and neurobehavioral effects in *Daphnia* [17]. Of particular concern, AceK has also been implicated in malignancy. Kim et al. demonstrated that AceK inhibited autophagic degradation of programmed death-ligand 1 (PD-L1) in hepatocellular carcinoma, thereby conferring resistance to T cell-mediated cytotoxicity. This suggests that AceK may promote liver cancer progression, as evidenced by experiments in mouse (RIL-175) and human (SK-Hep1) HCC cell lines treated with 1 mM AceK for 24 h [18].

As shown in Table 2, we summarized the possible effects of AceK on human health. The STOP Sugars NOW (Strategies To Oppose Sugars with Non-nutritive sweeteners Or Water) Trial is a pragmatic, randomized, open-label, crossover clinical study designed to assess whether replacing sugar-sweetened beverages (SSBs) with non-nutritive sweetened beverages (NSBs) or water improves metabolic outcomes and alters the gut microbiome. A total of 80 adults who were overweight or obese who habitually consumed SSBs were enrolled and completed a 4-week intervention period—continuing SSBs, substituting with NSBs, or substituting with water—with washout intervals between each phase. The complete results of this trial have not yet been fully published and are currently being presented at scientific meetings or undergoing journal submission [19]. With respect to cardiovascular diseases, a recent cohort study reported that AceK was associated with an increased risk of coronary heart disease [12], and patients with symptomatic carotid atherosclerosis were found to have elevated plasma levels of AceK and saccharin [20].

Overall, while some studies show no metabolic effects, others link AceK to impaired glucose tolerance, increased cardiovascular risk, dyslipidemia, atherosclerosis, and possible cancer-promoting effects. Further researches are needed to clarify its long-term safety.

**Table 2:** AceK-related human studies.

Category	Measured Parameter	Conclusion	Author, Year [Ref.]
RCT	Glycemic responses	Interference of oral glucose tolerance	Ayoub-Charette et al., 2023 [19]
Case control	Disease risks	Increased coronary heart disease risk	Debras, C., et al., 2022 [12]
Case control	Disease risks	Increase the risk of symptomatic carotid atherosclerosis	Stø, K., et al., 2023 [20]

RCT, randomized controlled trial.

### 1.3. Aspartame

Aspartame is an artificial sweetener that is commonly used in sugar-free and “diet” food and beverages. It is approximately 200 times sweeter than sucrose [21]. The ADI of aspartame is 40 mg per kilogram of body weight per day per the EFSA and 50 mg per kilogram of body weight per day per the U.S. FDA. It was first discovered in 1965 by James M. Schlatter and has been the subject of numerous safety evaluations since its approval by the U.S. FDA in 1981. In contrast to sucralose and AceK, aspartame is metabolized in the body and broken down in the small intestine by digestive enzymes into phenylalanine, aspartic acid, and methanol.

Aspartame has long been a subject of criticism and controversy, particularly regarding its alleged associations with cancer and other adverse health outcomes. Owing to its low-calorie property, aspartame has been widely used for weight management and glycemic control. In animal and cell-based experiments, aspartame has been reported to alter gut microbial composition, including increases in *Enterobacteriaceae* and *Clostridium leptum*, thereby impairing glucose tolerance in rats administered 5–7 mg/kg/day for 8 weeks [22,23]. In response to growing concerns about aspartame safety, the Ramazzini Institute (RI) initiated a series of large-scale toxicological studies in 1997 to evaluate its potential carcinogenicity. In one study, Sprague-Dawley rats were exposed to estimated daily doses of 4–5000 mg/kg body weight until natural death (~159 weeks). A positive dose–response relationship was observed, with the highest incidence of malignancies occurring in animals exposed to the highest doses of aspartame [24,25,26,27]. Although the RI findings were controversial due to questions regarding the accuracy of histopathological diagnoses, a subsequent re-examination of hematopoietic and lymphoid tumors using immunohistochemistry and morphological reclassification confirmed that 92% of the lesions in aspartame-exposed animals were indeed malignant [28]. This reanalysis underscored the urgency of re-evaluating aspartame’s health risks, particularly those associated with prenatal exposure [29]. Overall, the available animal evidence indicates dose-related tumorigenicity and highlights the necessity of reassessing aspartame safety. Long-term studies remain essential to confirm its carcinogenic risk and clarify its safety profile.

As shown in Table 3, we summarized the potential effects of aspartame on human health. Several human studies have reported that aspartame provides no beneficial effects on the regulation of blood glucose homeostasis [30,31,32,33,34]. Similarly, a randomized controlled trial involving 100 healthy, lean adults who consumed aspartame at doses of 350–1050 mg/day for 12 weeks found no significant changes in body weight [32]. Investigations into the effects of non-nutritive sweeteners (NNS), including aspartame, on body weight also demonstrated no differences in weight change between NNS groups and placebo or water groups [9,35]. With regard to cardiovascular outcomes, aspartame intake has been linked to an increased risk of cerebrovascular disease [12,36]. The potential association between artificial sweeteners and cancer has also attracted growing attention. A large prospective cohort study of 102,865 French adults (2009–2021), with exposure assessed through repeated 24 h dietary records and quantitative estimates of artificial sweetener intake over a median follow-up of 7.8 years, reported that aspartame and AceK consumption were associated with an elevated cancer risk [37]. However, this association may be confounded by underlying characteristics of consumers—such as a higher prevalence of obesity, metabolic disorders, or specific dietary patterns—rather than indicating a direct carcinogenic effect of the sweeteners themselves.

**Table 3:** Aspartame-related human studies.

Category	Measured Parameter	Conclusion	Author, Year [Ref.]
Case control	Glycemic responses	No significant effects	Bonnet, F., et al., 2018 [30]
Cohort	Glycemic responses	No significant effects	Bryant, C.E., et al., 2014 [31]
RCT	Glycemic responses	No significant effects	Higgins, K.A., R.V. Considine, and R.D. Mattes, 2018 [32]
RCT	Glycemic responses	No significant effects	Olalde-Mendoza, L. and Y.E. Moreno-González, 2013 [33]
RCT	Glycemic responses	No significant effects	Tey, S.L., et al., 2017 [34]
Case control	Disease risks	Higher risk of cerebrovascular disease	Debras, C., et al., 2022 [12]
Case control	Disease risks	Higher risk of cerebrovascular disease	Bernstein, A.M., et al., 2012 [36]
Case control	Disease risks	Increased cancer risk	Debras, C., et al., 2022 [37]
Meta-analysis	Body weight	No effect on body weight changes in the group of NNS	Rogers, P.J. and K.M. Appleton, 2021 [35]

RCT, randomized controlled trial; NNS, non-nutritive sweeteners.

### 1.4. Saccharin

Saccharin is one of the oldest artificial sweeteners, first discovered in 1879. The ADI of saccharin is 5 mg per kilogram of body weight per day by the U.S. FDA. Similar to sucralose and AceK, saccharin is rapidly absorbed in the small intestine after ingestion and excreted by prototype through the urine by the kidney, and more than 90% of absorbed saccharin is excreted within 24 h [38]. The small amount of saccharin that is not absorbed in the intestines is eliminated through feces.

An animal study demonstrated that long-term saccharin consumption did not significantly influence body weight, fat mass, or glucose metabolism, as compared with water intake [15]. However, saccharin has been the subject of considerable controversy due to early animal studies in rats, in which high dietary exposures—typically 5% saccharin in the diet (2–4 g/kg body weight/day)—were associated with potential cancer risk [39,40].

As shown in Table 4, we summarized the potential effects of saccharin on human health. Most studies have reported no significant association between saccharin consumption and blood glucose control [31,41]. Similarly, only limited effects on body weight reduction have been observed [15,35]. Substituting sugar with non-nutritive sweeteners, including saccharin, may represent the only favorable aspect of saccharin with respect to weight management [42]. In contrast, a randomized controlled trial with 12 weeks of saccharin intervention reported an incremental change in body weight [43]. Overall, these findings indicate limited metabolic benefits of saccharin. Regulatory authorities, including the U.S. FDA and the World Health Organization (WHO), have deemed saccharin safe for human consumption. Nevertheless, its health effects remain a matter of ongoing debate, and no global consensus has been established. Epidemiological evidence has suggested a significant association between saccharin intake and increased risks of mortality, particularly cardiovascular and cancer-related mortality, in diabetic and pre-diabetic populations [44]. Moreover, in a study including 15 patients with symptomatic carotid atherosclerosis, 18 patients with asymptomatic carotid atherosclerosis, and 15 healthy controls, elevated plasma saccharin levels (7.6 ± 6.8 ng/mL in symptomatic carotid atherosclerosis vs. 2.0 ± 2.2 ng/mL in healthy controls) were associated with greater carotid stenosis and higher weekly snack consumption, potentially implicating saccharin in the pathogenesis of cardiovascular disease [20,45]. Regarding malignancy, toxicological and epidemiological evidence has generally shown no association between saccharin or other NNS and overall cancer risk [46]. However, accumulating evidence from 12 observational studies (10 cohort, 2 case-control) involving 572,820 participants has indicated a potential association between saccharin intake and an increased risk of endometrial cancer [37,47,48]. Considering all the above, it can be inferred that possible associations with increased mortality and endometrial cancer warrant further investigation, especially in vulnerable populations.

**Table 4:** Saccharin-related human studies.

Category	Measured Parameter	Conclusion	Author, Year [Ref.]
Cohort	Glycemic responses	No significant effect	Bryant, C.E., et al., 2014 [31]
RCT	Glycemic responses	No significant effect	Orku, S.E., G. Suyen, and M. Bas, 2023 [41]
Meta-analysis	Body weight	No significant effect	Rogers, P.J. and K.M. Appleton, 2021 [35]
RCT	Body weight	May increase body weight	Higgins, K.A. and R.D. Mattes, 2019 [43]
Case control	Disease risks	Increase the risk of mortality, particularly cardiovascular disease mortality and cancer mortality	Gao, Y., et al., 2024 [44]
Case control	Disease risks	Associated with increased degree of carotid stenosis	Stø, K., et al., 2023 [20]
Systemic review	Disease risks	No evidence of cancer risk associated	Pavanello, S., et al., 2023 [46]
Meta-analysis	Disease risks	May be associated with endometrial cancer	Li, H., et al., 2024 [48]

RCT, randomized controlled trial.

### 1.5. Stevioside

Stevioside is derived from *Stevia rebaudiana*, native to South America where it has been used for its sweet properties for hundreds of years. It is a glycoside, 50–350 times sweeter than sucrose, chemically composed of steviol with attached glucose molecules. The ADI of steviol equivalents is 4 mg per kilogram of body weight per day per the EFSA and Joint FAO/WHO Expert Committee on Food Additives (JECFA), whereas stevioside is not permitted for use as a sweetener by the U.S. FDA. The ADI is expressed in steviol equivalents since stevioside and related compounds (such as rebaudioside A) are metabolized into steviol in human bodies [49]. Stevioside is not absorbed directly in the upper gastrointestinal tract; however, it is broken down into steviol by gut microbiota while it reaches the colon. The steviol, hydrolyzed from stevioside by gut bacteria, is absorbed into the bloodstream and transported to the liver. In the liver, steviol undergoes glucuronidation, forming steviol glucuronide, which is water-soluble and easier to be excreted in urine. The majority (90%) of steviol glucuronide is excreted unchanged via urine within 24–48 h, whereas a small portion of steviol is excreted in feces [50].

With regard to potential health effects, animal studies in rats have demonstrated that stevioside exerts hypolipidemic effects, characterized by reductions in cholesterol, triglycerides, and LDL-c, along with increases in HDL-c [51,52,53]. Furthermore, several in vitro studies have intriguingly suggested that stevioside possesses antitumor properties against bladder, colon, and breast cancer cells [54,55,56,57].

As shown in Table 5, we summarized the reported effects of stevioside in human studies. Stevioside appears to exert beneficial effects on glucose metabolism, particularly when used as a sugar substitute in younger, non-diabetic, and overweight/obese populations [58]. Several human studies have assessed this effect through single-meal evaluations, comparing reduced-sugar and calorie meals containing steviol glycosides with full-sugar, calorie-matched meals. In randomized controlled trials, one involving 31 healthy adults across three test days and another involving 12 patients with type 2 diabetes, significant reductions in postprandial blood glucose levels were observed when steviol glycosides were incorporated into reduced-sugar/calorie meals or administered as supplements in both healthy and diabetic individuals [59,60]. With respect to lipid regulation, a study in 20 women with elevated serum cholesterol demonstrated that daily consumption of 20 mL stevia extract (prepared from 3.3 g of stevia leaves) diluted in 200 mL water for one month significantly reduced cholesterol, triglyceride, and LDL-c levels, while increasing HDL-c [61]. Collectively, these findings suggested that stevioside may contribute to improved metabolic regulation. Overall, its favorable safety profile and natural origin highlight stevioside as a promising sugar substitute; however, further long-term clinical studies are warranted to confirm these benefits.

**Table 5:** Stevioside-related human studies.

Category	Measured Parameter	Conclusion	Author, Year [Ref.]
Meta-analysis	Glycemic responses	May provide beneficial effects on glucose metabolism	Bai, X., et al., 2024 [58]
RCT	Glycemic responses	Significant decrease in postprandial blood glucose levels	Anton, S.D., et al., 2010 [59]
RCT	Glycemic responses	Significant decrease in postprandial blood glucose levels	Gregersen, S., et al., 2004 [60]

RCT, randomized controlled trial.

### 1.6. Erythritol

Erythritol is a naturally occurring polyol (sugar alcohol) with the chemical formula C_4_H_10_O_4_. It occurs naturally in small amounts in various fruits (e.g., grapes, melons, and pears), mushrooms, and fermented foods (e.g., soy sauce, wine, and cheese). Industrially, it is produced by the fermentation of glucose using osmophilic yeasts, mainly *Moniliella pollinis* or *Trichosporonoides megachiliensis* [62]. Erythritol exhibits a relative sweetness of approximately 60% to 80% that of sucrose, providing a comparable organoleptic profile with reduced caloric load. After ingestion, erythritol is rapidly absorbed in the small intestine via passive diffusion due to its small molecular weight. Unlike other polyols (e.g., sorbitol or xylitol) that undergo partial fermentation in the colon, erythritol is poorly metabolized by gut microbiota. About 90% is excreted unchanged in urine within 24 h; only about 10% reaches the colon and may be fermented slightly with minimal gas production. According to EFSA, its energy value is estimated at approximately 0.2 kcal per gram, primarily because more than 90% of ingested erythritol is absorbed in the small intestine and subsequently excreted unchanged in the urine, resulting in minimal caloric contribution. Erythritol does not have an officially established ADI. Erythritol has been granted Generally Recognized as Safe (GRAS) status by both the U.S. FDA and EFSA, reflecting its established safety for use as an LCS in various food and beverage applications. EFSA’s Scientific Committee on Food (SCF) concluded that erythritol’s absorption and rapid excretion make it unlikely to pose toxicological concerns at reasonable levels of intake, so a numerical ADI was not deemed necessary [63].

In an animal study with rats, erythritol administration resulted in significant reductions in glucose intolerance, blood glucose, and hemoglobin A1c (HbA1c) levels [64]. Moreover, erythritol may be associated with an increased risk of major adverse cardiovascular events. Mechanistic investigations further showed that erythritol at physiological concentrations enhanced platelet reactivity in vitro—manifested by increased aggregation, intracellular Ca^2+^ flux, P-selectin expression, and GP IIb/IIIa activation—and promoted platelet adhesion and thrombosis formation in mice [65].

As shown in Table 6, we summarized the possible effects of erythritol on human health. Erythritol does not increase plasma glucose or insulin levels, making it suitable for diabetic populations [66,67,68]. Munro et al. conducted an interpretive review of clinical findings and concluded that repeated daily consumption of erythritol at doses up to ~1 g/kg body weight for several weeks does not significantly affect markers of glycemic control, including HbA1c, even among individuals with impaired glucose metabolism [63]. With regard to body weight, only a limited number of clinical trials have directly assessed erythritol’s effects [66]. In one small study, 12 healthy men consumed 1 g/kg/day of erythritol for 7 days, with no significant changes in body weight observed [69]. Another small trial involving seven patients with type II diabetes reported an average weight reduction of ~2 kg after daily intake of 20 g erythritol for 2 weeks; however, this finding did not reach statistical significance, as the effect was primarily driven by three individuals [70]. Concerning lipid metabolism, a randomized controlled trial in 21 healthy adults demonstrated that erythritol did not significantly alter the blood lipid profile, including total cholesterol, LDL-c, HDL-c, and triglycerides [71]. These studies suggested that erythritol is a well-tolerated LCS with minimal impact on glucose and lipid metabolism. Recently, however, concerns have been raised regarding its cardiovascular safety. An untargeted metabolomics study in patients undergoing cardiac risk assessment (discovery cohort, n = 1157) revealed that higher plasma erythritol levels were strongly associated with an increased 3-year risk of major adverse cardiovascular events. In a pilot human-intervention study (n = 8), ingestion of 30 g erythritol rapidly elevated plasma concentrations to more than 1000-fold above baseline, remaining above pro-thrombotic thresholds for over 48 h [65]. Thus, further research is warranted to evaluate its long-term safety in human populations.

**Table 6:** Erythritol-related human studies.

Category	Measured Parameter	Conclusion	Author, Year [Ref.]
Cohort	Glycemic responses	Does not increase plasma glucose or insulin levels	Noda, K., K. Nakayama, and T. Oku, 1994 [67]
RCT	Glycemic responses	Does not increase plasma glucose or insulin levels	Wölnerhanssen, B.K., et al., 2016 [68]
Cohort	Body weight	No significant changes in body weight	Tetzloff, W., et al., 1996 [69]
Cohort	Body weight	No significant changes in body weight	Ishikawa, M., et al., 1996 [70]
RCT	Serum lipid profile	No significant effects on lipid profile	Teysseire, F., et al., 2023 [71]
Cohort	Disease risks	Strongly associated with a higher 3-year risk of major adverse cardiovascular events	Witkowski, M., et al., 2023 [65]

RCT, randomized controlled trial.

### 1.7. Xylitol

Xylitol is a naturally occurring five-carbon sugar alcohol (a polyol) with the chemical formula C_5_H_12_O_5_. It is found in small amounts in various fruits and vegetables, such as plums, strawberries, and cauliflower, and is commercially produced by the hydrogenation of xylose derived from birch wood or corncobs. Xylitol is approximately as sweet as sucrose but provides about 40% fewer calories (~2.4 kcal/g) [72]. To date, no numerical ADI for xylitol has been established by JECFA or EFSA due to its generally recognized safety when used in normal dietary amounts. Nonetheless, doses exceeding 20–40 g/day are more likely to cause laxative effects; therefore, divided intake is recommended to improve gastrointestinal tolerance [73]. Approximately 50% of ingested xylitol is absorbed in the small intestine via passive diffusion. Absorbed xylitol undergoes hepatic metabolism, entering the pentose phosphate pathway where it is converted into glucose and glycogen. The remaining unabsorbed fraction is fermented by colonic microbiota, which may lead to gastrointestinal discomfort (e.g., bloating, flatulence, osmotic diarrhea) if consumed in excessive amounts [74]. Unlike sucrose, xylitol has minimal effects on postprandial glucose and insulin levels, making it suitable for individuals with impaired glucose tolerance or diabetes [75]. Although xylitol provides approximately 40% fewer calories than sucrose (2.4 kcal/g vs. 4 kcal/g), there is currently insufficient high-quality clinical evidence to support a significant impact of xylitol consumption on long-term body weight reduction. Theoretically, partial substitution of sucrose with xylitol can contribute to overall caloric reduction, potentially aiding in weight management when combined with other dietary and lifestyle interventions [74]. However, excessive intake may lead to gastrointestinal disturbances due to partial fermentation by colonic microbiota. Therefore, such laxative effects are not a safe or sustainable weight control strategy [76].

On the aspect of the influence on the blood lipid profile, human data are limited and inconsistent at present. Several animal studies suggested that xylitol may have favorable effects on lipid metabolism. An animal study showed that plasma total cholesterol levels were significantly lower when fed with high dose xylitol (1.5 g/kg body weight/day of dietary) [77]. On the other hand, another animal study revealed that no significant changes in hepatic or serum triglycerides and cholesterol were seen with low/medium (40–194 mg/kg body weight per day) xylitol in normal diet mice [78]. Also, elevated plasma xylitol accelerated arterial thrombus formation in mice [79]. In aggregate, xylitol is an LCS that exerts minimal impact on glucose metabolism and may confer beneficial effects on lipid profiles.

As shown in Table 7, we summarized the possible effects of xylitol on human health. Regulatory assessments by WHO and EFSA, based on long-term safety data, found no carcinogenicity or chronic disease risks, approving xylitol as safe. However, in a June 2024 study in the European Heart Journal (by Cleveland Clinic researchers) with two large human cohorts (n ≈ 3300), higher fasting plasma xylitol levels were significantly associated with an increased 3-year risk of major adverse cardiovascular events (MACE), including heart attack, stroke, or death. Human blood studies showed that physiological and postprandial levels of xylitol enhanced platelet reactivity, making blood more prone to clot [79]. The observed association between elevated plasma xylitol levels and heightened cardiovascular risk highlights the importance of further studies to clarify its long-term safety profile.

**Table 7:** Xylitol-related human studies.

Category	Measured Parameter	Conclusion	Author, Year [Ref.]
Cohort	Glycemic responses	Minimal effects on postprandial glucose and insulin levels	Natah, S.S., et al., 1997 [75]
Cohort	Disease risks	Associated with an increased 3-year risk of major adverse cardiovascular events	Witkowski, M., et al., 2024 [79]

### 1.8. Maltitol

Maltitol is a hydrogenated disaccharide polyol (sugar alcohol) derived from maltose, with the chemical formula C_12_H_24_O_11_. It provides approximately 75–90% of the sweetness of sucrose, while delivering fewer calories (approximately 2.1–2.4 kcal/g, compared to 4 kcal/g for sucrose). Due to its similar sweetness profile and functional properties, maltitol is widely used as a sugar substitute in various sugar-free or reduced-calorie products, such as chocolates, baked goods, chewing gum, and confectionery [80]. About 40–50% of ingested maltitol is absorbed in the small intestine via passive diffusion, then metabolized primarily in the liver to glucose and sorbitol, and further to fructose and glucose-6-phosphate. The remaining 50–60% reaches the colon, where it undergoes fermentation by gut microbiota, producing short-chain fatty acids (SCFAs), hydrogen, and methane gases [74]. Maltitol is classified as GRAS by the U.S. FDA and approved by EFSA for use in a variety of food products. No numerical ADI has been set by JECFA due to its generally low toxicity, but excessive intake may lead to gastrointestinal discomfort. Doses exceeding 40–50 g/day in adults may cause osmotic diarrhea, bloating, or flatulence due to colonic fermentation [81].

As shown in Table 8, we summarized the possible effects of maltitol on human health. A study showed that following an acute oral dose of 50 g maltitol, the glycemic and insulinemic responses were significantly lower, as compared with an equivalent dose of glucose or sucrose. In the chronic phase, daily intake of maltitol (10 g three times daily for five consecutive days) produced no significant changes in blood glucose or insulin levels relative to the same dose of sucrose [82]. These findings confirmed that maltitol elicits a reduced postprandial glycemic and insulinemic response, making it suitable for individuals who require blood glucose management, such as people with diabetes. To date, no randomized controlled trial has examined maltitol in isolation to determine if it leads to weight loss. The evidence relies primarily on indirect inferences from studies on polyols as a group or sugar substitutes more broadly. Overall, the evidence indicated that LCS, including polyols such as maltitol, exert modest or neutral effects on body weight when they are used as true substitutes for caloric sugars rather than simply added to the diet [83]. Collectively, these findings indicated that maltitol elicits lower glycemic and insulinemic responses than sucrose, supporting its suitability for blood glucose management, although evidence for its effects on weight loss remains limited. Meanwhile, animal and human studies have provided no indication that maltitol possesses carcinogenic, mutagenic, or genotoxic properties. The main health issue with maltitol is gastrointestinal discomfort when consumed in high doses (typically >40–50 g/day for adults). Unabsorbed maltitol can be fermented in the colon, producing gas and osmotic effects, leading to bloating, flatulence, or mild laxative effects [81].

**Table 8:** Maltitol-related human studies.

Category	Measured Parameter	Conclusion	Author, Year [Ref.]
Cohort	Glycemic responses	Significantly lower blood sugar when following an acute oral dose	Secchi, A., et al., 1986 [82]
Meta-analysis	Body weight	Modest or neutral effects on body weight when they are used as true substitutes for caloric sugars rather than simply added to the diet	Miller, P.E. and V. Perez, 2014 [83]

### 1.9. Sorbitol

Sorbitol is a versatile sugar alcohol used extensively in food, pharmaceuticals, and cosmetics. The ADI for sorbitol has also not been specifically established. Although there is no official ADI and sorbitol is GRAS, excessive consumption of sorbitol may cause gastrointestinal issues, including bloating, gas, and diarrhea. A study of 70 healthy young adults suggested that consuming over 20–50 g of similar sugar alcohol, including erythritol and xylitol, per day may lead to laxative effects [73,74]. EFSA regulated that foods containing more than 10% sorbitol must carry a warning label in Europe. Its ability to provide sweetness without contributing significantly to caloric (around 2.6 kcal/gram) intake makes it popular for sugar-free and diabetic-friendly products. Sorbitol is slowly absorbed in the small intestine via passive diffusion. Due to its poor absorption, a portion of sorbitol remains in the intestines, where it can be fermented by gut bacteria, leading to gas production or retaining water in the intestine, leading to a laxative effect. Once absorbed, sorbitol is primarily metabolized in the liver via the polyol pathway and converts into fructose by sorbitol dehydrogenase.

Sorbitol may contribute to weight management as an LCS substitute; however, human studies directly linking its use to long-term weight control remain limited. In animal models, sorbitol has been shown to inhibit intestinal glucose absorption, enhance glucose uptake in muscle tissue, delay gastric emptying, and reduce blood glucose levels in both normoglycemic and diabetic rats treated with 0.4 g/kg sorbitol in combination with 2 g/kg glucose [84]. These findings suggested that sorbitol has potential as an anti-hyperglycemic sweetener for individuals with diabetes. However, a study indicated that long-term sorbitol consumption may induce glucose intolerance in mice through alterations in the gut microbiota [85].

As shown in Table 9, we summarized the potential effects of sorbitol on human health. In a clinical study, daily intake of 10 g sorbitol for one month produced no significant changes in blood glucose levels [86]. Sorbitol is, however, slowly absorbed in the small intestine, and excessive consumption (≥20–50 g/day) may lead to gastrointestinal discomfort and laxative effects. In severe cases, gastrointestinal complications such as perforated colonic ulcers, bleeding, ischemic colitis, and colonic necrosis have been reported [87]. Collectively, these findings suggested that sorbitol may provide glycemic benefits; nevertheless, the limited human evidence and the gastrointestinal risks associated with high doses warrant cautious use. Further investigations are required to elucidate its long-term effects on glucose metabolism and microbial composition.

**Table 9:** Sorbitol-related human studies.

Category	Measured Parameter	Conclusion	Author, Year [Ref.]
Cohort	Glycemic responses	No significant effects on blood glucose levels	Ellis, F.W., and JC, Jr Krantz., 1941 [86]

## 2. Possible Mechanisms of LCS in the Regulation of Metabolic Homeostasis

### 2.1. Receptor-Mediated Pathway

LCS are ligands of the sweet taste receptor. The sweet taste receptors (STRs), including taste receptor type 1 member 2 (TAS1R2) and taste receptor type 1 member 3 (TAS1R3), are G-protein-coupled receptors (GPCRs) that detect sweet stimuli on the tongue bud and in the gut, pancreas, and brain. STRs are accompanied by G-proteins, such as gustducin (Gt), Gs, and Gi/o, which regulate different metabolic processes [88]. Also, the STR plays a significant role in carbohydrate metabolism. Typically, the regulation of carbohydrates in the body relies on the pancreas detecting blood glucose levels, transporting and oxidizing glucose, and producing adenosine triphosphate, which subsequently triggers insulin release to maintain glycemic balance. However, the STR also contributes to several carbohydrate-related processes, including pre-absorptive cephalic phase insulin release [89], regulation of glucagon-like peptide 1 (GLP-1) secretion by enteroendocrine cells [90,91], upregulation and translocation of sodium-glucose cotransporter (SGLT) and glucose transporter 2 (GLUT2) in the intestine [92,93,94,95], and direct stimulation of insulin secretion from the pancreas [96,97]. Sweeteners interfere with glucose metabolism through their interaction with STR and downstream signaling pathways in gut enteroendocrine cells and pancreatic β-cells.

Activation of TAS1R2/TAS1R3 leads to upregulation of SGLT1 on gut enteroendocrine cells, further increasing glucose absorption. However, not all sweeteners activate the sweet taste receptor on enteroendocrine cells (EECs) [98]. When LCS bind to the STR, Gα-gustducin is then coupled, leading to increased intracellular cyclic adenosine monophosphate (cAMP) and the activation of phospholipase C [99]. This further results in the release of calcium (Ca^2+^) from intracellular stores to trigger downstream effects, including SGLT-1 upgrade expression; the promotion of GLUT2 translocation to the apical membrane, further increasing glucose uptake; and an increase in GLP-1 secretion and disruption of normal glucose-dependent insulinotropic polypeptide (GIP) signaling, potentially desensitizing insulin responses. TAS1R2/TAS1R3 is also present in pancreatic β-cells, and activation by sweeteners can enhance insulin secretion [90].

Oxidative stress is a key factor linking metabolic disorders such as obesity, diabetes, and hypertension. Reactive oxygen species (ROS) disrupt the insulin-signaling pathway to impair glucose uptake, leading to insulin resistance. In a normal insulin-signaling pathway, when insulin binds to the insulin receptor (IR), it triggers the autophosphorylation of IR and further phosphorylates insulin receptor substrate (IRS) at tyrosine residues. IRS-1 and IRS-2 activate phosphoinositide 3-kinase (PI3K) to recruit Akt, which plays a major role in glucose metabolism. ROS promote oxidation of cysteine residues in IR to change its conformation and impair the autophosphorylation of IR. As a result, IRS proteins are not effectively activated, and Akt phosphorylation is reduced, leading to reduced GLUT4 translocation consequently. This results in decreased glucose uptake into cells, leading to hyperglycemia [100].

An animal study revealed that repeated stimulation of STRs by sucralose causes increases in ROS [101], indicating the increase in ROS generation might participate in LCS-induced adverse effects. In addition, sucralose promotes ROS accumulation and enhances adipogenesis in human adipose tissue-derived mesenchymal stromal cells [101]. Also, the increment in ROS might further activate extracellular-regulated protein kinases (ERK1/2) pathways to disrupt insulin signaling [102]. These effects may contribute to insulin resistance and metabolic dysregulation with long-term use.

STRs in the brain and gut influence metabolic responses by modulating neural signals to the hypothalamus. AceK and sucralose activate brain reward pathways, potentially leading to increased food intake and altered glucose metabolism [103,104,105]. In contrast, steviol and steviol glucuronide exhibit anti-hyperglycemic effects by activating glucose-induced insulin secretion to enhance pancreatic β-cell function [106]. They may be beneficial for diabetes management in the future. Moreover, in addition to STRs, a previous study indicated that stevioside lowers plasma glucose via the opioid mu opioid receptor in animals in a dose-dependent manner [107].

Sorbitol is slowly converted into fructose and glucose in the liver and pancreatic cells via the sorbitol–fructose metabolic pathway. Since fructose can enhance insulin secretion via STRs [97], sorbitol may have an indirect effect once metabolized into fructose. However, unlike fructose, sorbitol does not rapidly stimulate insulin secretion due to its slower metabolism.

### 2.2. LCS Alters Gut Microbiome

The gut microbiome is a critical modulator of host metabolism and T2DM risk. Emerging evidence indicates that alterations in the gut microbial composition—particularly a reduced Bacteroidetes-to-Firmicutes (B/F) ratio—are associated with increased energy harvest, metabolic dysregulation, chronic low-grade inflammation, and insulin resistance, all of which contribute to the development of T2DM. Key microbial metabolites, such as SCFAs, exert protective effects on gut barrier integrity and immune modulation, whereas elevated levels of branched-chain amino acids (BCAAs) are implicated in impaired insulin signaling. Experimental and clinical studies further demonstrate that modulation of the microbiome through dietary interventions, bariatric surgery, microbial transplantation, or pharmacological agents such as metformin can ameliorate metabolic dysfunction [108]. These findings underscore the significant impact of the gut microbiome on human health. However, recent studies have indicated that LCS may alter the composition and function of the gut microbiome, thereby potentially influencing host health.

Sucralose is poorly absorbed, and 85% reaches the colon unchanged, where it can interact with gut bacteria. However, studies revealed contradictory results. A randomized clinical trial (7 days) found no effect on gut microbiota and body weight; glycemic control and insulin resistance were not affected as well [4]. Another study (10 weeks) found minor reductions in *Lactobacillus acidophilus* and an increase in *Blautia coccoides*. Elevated insulin levels and increased glucose response (higher area under the curve for glucose during OGTT) was noted after sucralose intake. This suggests that sucralose may contribute to insulin resistance over time according to the study [109]. A recent study found that consumption of sucralose for two weeks significantly altered gut microbiota and increased glycemic response. Functional pathway changes were also noted in the study. Sucralose increased tricarboxylic acid cycle activity and reduced purine metabolism, which correlated with higher glycemic responses [45]. In addition, activation of TAS1R3 by sucralose promotes gut inflammation by activating mammalian target of rapamycin (mTOR) and suppressing peroxisome proliferator-activated receptor (PPAR)-γ, leading to increased inflammatory cytokines and intestinal barrier dysfunction. Wild-type mice had an overgrowth of inflammatory bacteria, including *Enterobacteriaceae* (associated with gut dysbiosis and inflammatory bowel disease), *Paraprevotellaceae*, and *Prevotella* (linked to gut inflammation). In contrast, Tas1r3 knockout mice had a significant increase in beneficial, butyrate-producing bacteria, such as *Lachnospiraceae*, *Butyrivibrio*, *Roseburia*, *Faecalibacterium*, and *Butyricicoccus*. Since butyrate is crucial for maintaining gut health and reducing inflammation, activation of TAS1R3 may result in dysbiosis of gut microbiota [110].

High doses of sucralose alter the structure of the T cell membrane, reducing membrane fluidity to impair T cell responses, particularly in CD8^+^ T cells. These changes make it harder for T cells to form effective immune synapses. In addition, T cells rely on calcium signaling for activation, and sucralose-treated T cells show reduced intracellular calcium release, leading to weakened immune activation [111]. In summary, exposure to sucralose may reduce the efficiency of T cell receptor signaling, which is essential for T cell activation and further results in gut microbiota dysbiosis. AceK is rapidly absorbed and excreted unchanged in urine; however, a human study found no significant differences in gut microbiota between AceK consumers and non-consumers [112].

Aspartame is broken down in the small intestine; therefore, aspartame itself might not be directly exposed to the gut microbiota in its intact form, whereas its metabolites may influence gut bacteria and metabolism; however, clinical studies revealed mixed results. A controlled trial found no effects on gut microbiota or metabolic markers after 2 weeks of aspartame consumption [113], whereas another trial showed that aspartame decreases the abundance of *Porphyromonas* and *Prevotella nanceiensis* in the oral microbiota.

Saccharin is partially absorbed, with some remaining in the gut, where it interacts with microbiota. A small human study (6 days, 5 mg/kg/day) demonstrated that four out of seven participants developed glucose intolerance, and their gut microbiota changed significantly, increasing *Bacteroides fragilis* and *Weissella cibaria* and decreasing *Candidatus Arthromitus*. Fecal microbiota transplantation from these human participants to germ-free mice resulted in similar glucose intolerance [114]. A recent study showed a significant increase in *Prevotella copri* in the saccharin group, accompanied with higher glycemic responses. Functional pathway changes with an increased uridine monophosphate (UMP) biosynthesis pathway were also noted. The clinical outcome of the study revealed that saccharin raised a glycemic response compared to glucose (*p* = 0.042) and no supplement control groups (*p* = 0.018) [45].

There is limited research on the effects of stevioside on human gut microbiota. A recent 12-week human study found that regular stevia consumption did not significantly alter the composition of the gut microbiota [115]. Sorbitol has notable effects on the human gut microbiota. A study indicated that extended sorbitol consumption could decrease the relative abundances of beneficial bacteria such as *Bifidobacterium* and certain members of the *Lachnospiraceae* family, while increasing the presence of genera like *Helicobacter* and *Prevotella*. These microbial shifts were associated with the development of glucose intolerance in the study subjects [85].

## 3. Conclusions

LCS are widely used as sugar alternatives in food and beverages, providing sweetness without the caloric burden of sucrose. However, while LCS serve as useful sugar substitutes, their long-term health effects remain a subject of debate (Table 10). While some sweeteners, such as stevioside, may provide health benefits, others, particularly certain artificial sweeteners, raise concerns regarding metabolic disturbances, cardiovascular risk, possible associations with cancer, and gut microbiota changes. More long-term, large-scale clinical studies are needed to fully understand their implications for human health. Until then, moderation remains key, and consumers should make informed choices based on their health needs and dietary preferences.

**Table 10:** Comparative summary of each LCS.

Low-Calorie Sweeteners	Current Consumption	Summary
Sucralose	Most frequently	May impair insulin sensitivity, alter gut microbiota, and affect liver and lipid metabolism
AceK	Most frequently	May associate with impaired glucose tolerance, cardiovascular risk, and possible cancer-promoting effects
Aspartame	Declining in new products but still widely present	May impair glucose tolerance, alter gut microbiota, and increas risks of cardiovascular disease and cancer
Saccharin	Less	Limited metabolic benefits but rising concerns about its potential effects on cardiovascular health and cancer risk
Stevioside	Rising	Potential metabolic benefits, including improved glycemic control and lipid regulation, with preliminary evidence suggesting antitumor properties
Erythritol	Rising	Minimal impact on glucose and lipid metabolism, but may increase cardiovascular risk
Xylitol	Commonly consumed in daily life, particularly in chewing gum and toothpaste	Minimal effects on glucose metabolism, potential lipid benefits, but may increase cardiovascular risk
Maltitol	Widely present in food formulations but lacking specific statistical data	Reduced glycemic effects, no known toxicological risks, but high doses may cause gastrointestinal discomfort
Sorbitol	Widely present in food formulations but lacking specific statistical data	Potential glycemic benefits, but limited human data and gastrointestinal risks at high doses
